# Role of NOD2/*CARD15 *in coronary heart disease

**DOI:** 10.1186/1471-2156-8-76

**Published:** 2007-11-02

**Authors:** Nour Eddine El Mokhtari, Stephan J Ott, Almut Nebel, Arne Schäfer, Philip Rosenstiel, Matti Förster, Michael Nothnagel, Rüdiger Simon, Stefan Schreiber

**Affiliations:** 1 Department of Cardiology, University Hospital Schleswig-Holstein, Kiel, Germany; 2 Institute of Clinical Molecular Biology, Christian-Albrechts-University, Kiel, Germany; 3 Department of General Internal Medicine, University Hospital Schleswig-Holstein, Kiel, Germany; 4 Institute of Medical Informatics and Statistics, University Hospital Schleswig-Holstein, Kiel, Germany

## Abstract

**Background:**

Bacterial DNA has been repeatedly detected in atheromatous lesions of coronary heart disease (CHD) patients. Phylogenetic signatures in the atheroma lesions that are similar to those of bacterial biofilms on human barrier organs, including the respiratory or gastrointestinal tract, raise the question of a defective barrier function in CHD. NOD2 plays a major role in defense against bacterial invasion. Genetic variation in the *CARD15 *gene, which encodes NOD2, was previously shown to result in a barrier defect that causes chronic inflammatory disorders (e.g. Crohn disease). In the present study, we investigated the possible involvement of NOD2/*CARD15 *in the pathology of CHD by *i) *analyzing the local expression of NOD2 in atherectomy versus healthy tissue (n = 5 each) using histochemical immunofluorescence and *ii) *by testing the three major functional *CARD15 *variants (R702W, G908R and 1007fs) for association with early-onset CHD in 900 German patients and 632 healthy controls.

**Results:**

In atherectomy tissue of CHD patients, NOD2 was detected in inflammatory cells at the luminal sides of the lesions. However, the allele and genotype frequencies of the three major *CARD15 *polymorphisms did not differ between CHD patients and controls.

**Conclusion:**

The NOD2 up-regulation in atheroma lesions indicates an involvement of this protein in the pathology of CHD. Although NOD2 could be important in local immune response mechanisms, none of the analyzed *CARD15 *variants seem to play a significant role in the etiology of CHD.

## Background

Over the last years, it has become apparent that inflammation is a driving factor in the pathophysiology of coronary heart disease (CHD). Immune cells infiltrate the coronary artery lesion, and their mediator molecules aggravate progression and activation of the atherosclerotic lesions [1-4]. The importance of elevated C-reactive protein (CRP) as a negative prognostic marker of disease progression underlines the role of inflammation as a pathogenic factor [5]. There is increasing evidence from sero-epidemiological and animal studies that bacterial infection may be involved in the pathophysiology of the local chronic inflammatory process underlying atherosclerosis [6-9]. The initial observation of a specific infection with a single pathogen, e.g. *Chlamydia pneumoniae*, was increasingly challenged by new experimental findings demonstrating the presence of a wide spectrum of bacteria in the atherosclerotic plaque [10]. The variety and composition of bacterial species are compatible with the bacterial flora of human mucosal barriers [10,11], such as the oral cavity, the gastrointestinal, and the respiratory tract. As bacteria can translocate into the blood or lymph system from barrier organ surfaces, a secondary colonization of the plaques has been suggested. Lethiniemi *et al*. recently proposed that the destroyed surface of atheromas might act as mechanical sieves collecting bacteria from the circulation [11]. Impairment of mucosal barriers could therefore be a key factor in the pathogenesis of CHD as it allows bacteria to invade from body surfaces into systemic circulation and to colonize the atherosclerotic lesion.

Genetic factors that influence barrier function and immunological host responses to the microbial challenge may be determinants of susceptibility to CHD. Mutations in genes encoding components of the innate immune system that are involved in bacterial defense have been previously linked to the pathophysiology of CHD, as demonstrated for toll-like receptors (TLR) [12,13]. The TLR family represents a class of membrane-bound pattern-recognition receptors (PRRs) capable of detecting microbial components and products. Importantly, TLRs and other PRRs recognize not only exogenous but also endogenous ligands, for example the scavenger receptors SR-A and CD36 as well as oxidatively modified LDL (ox-LDL) or enzymatically-modified LDL(e-LDL) which are major players in the pathogenesis of atherosclerosis [14,15]. The relevance of TLR function in atherosclerosis is supported by the observation that a deletion of the MyD88 gene, encoding a pivotal adaptor molecule in the TLR signalling pathway, inhibits atherosclerosis in *apoE*^-/- ^mice [16].

Georges *et al*. investigated the impact of pathogen burden in patients with CHD in relation to systemic inflammation and variation in cytokine genes [17]. The study showed that CHD is associated with heightened systemic antibody response to different pathogens. Interestingly, the association between CHD and pathogen burden was modulated by the IL6 -174C/G polymorphism, which would result in a down-regulated immune activation and lower CRP levels [17]. More recently, NOD1 and NOD2, which are members of a nucleotide-binding- oligomerization-domain-leucin-rich-repeat (NOD-LRR) family of proteins, were described as important intracellular PRRs for bacterial cell wall proteins. NOD1 and NOD2 regulate activation of nuclear factor κB (NF-κB) in human fibroblast and aortic endothelial cell lines in response to *Chlamydia pneumoniae*, one of the most common bacterial species detected in atherosclerotic plaques [18]. Furthermore, endothelial cells upregulate *CARD15 */NOD2 in response to stimuli known to promote expression of the gene [19]. Fillon *et al*. reported that bacterial components interact with NOD2 in the cytoplasma of endothelial cells and cardiomyocytes [20].

Variants in *CARD15*, the gene encoding the NOD2 protein, are associated with Crohn disease (CD), a chronic inflammatory disorder of the human intestine [21], asthma and atopic eczema [22,23]. The three coding polymorphisms R702W, G908R and 1007fs in *CARD15 *all affect the leucin-rich repeat sensor domains of the NOD2 protein and are the main genetic risk determinants for CD. They are not in linkage disequilibrium (LD) with each other (r^2 ^values range between 0 and 0.002) and capture most of the positional information at the locus [24]. The variant protein fails to sense muramyl dipeptide (MDP), a structure of bacterial cell walls, that normally triggers activation of NFκB [25-27]. The exact nature of the disease mechanism is not completely clear. Transgenic mice that are either deficient for *CARD15 *or carry the most common CD susceptibility allele, 1007fs, exhibited a strongly disturbed activation of NF-κB activation in response to MDP [26,27]. Moreover, NOD2 elicits a complex protective cellular program due to constitutive expression of a subgroup of microbial peptides, known as α- and β-defensins in humans, that have been shown to be involved in the host response to commensal bacteria [26,28].

In this study, atherosclerotic coronary lesions from patients with CHD were examined for local expression of NOD2 by immunostaining. As control material coronary vessels from heart beating donors were used that showed no macroscopic alterations of the vessel wall. Furthermore, we investigated the occurrence and frequencies of the three most common polymorphisms in the *CARD15 *gene that are associated with mucosal barrier dysfunction (R702W, G908R and 1007fs) in a sample of patients with CHD (n = 900) and compared the results to those obtained from healthy controls (n = 632).

## Results

### Expression of NOD2 in coronary arteries is mainly localized in macrophages

**Figure 1:**
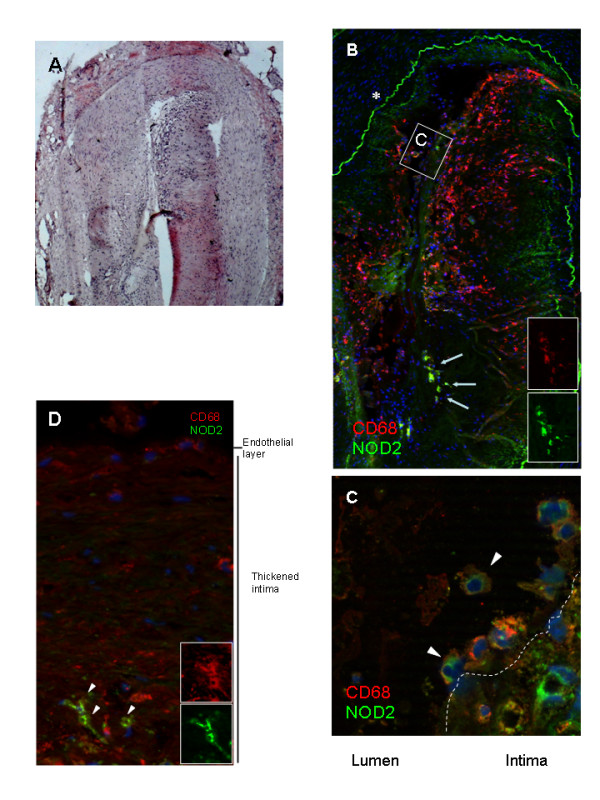
NOD2 detection in coronary atherosclerotic lesions by immunofluorescence. Panel **A **depicts an overview of a coronary artery section with a plaque at low magnification (HE staining, original magnification ×5). The same section has been stained for NOD2 (FITC, green) and the macrophage marker CD68 (Cy3, red) in **B**. The asterisk shows the strong autofluorescence of the fragmented external elastic membrane, the arrows depict NOD2 positive macrophages in deeper layers of the arterial wall (original magnification, ×10). The two inserts on the lower right hand side show double positive cells for CD68+ and NOD2. Panel **C **:is a higher power view of NOD2+/CD68+ cells, which can be found in the vicinity of the vessel lumen in the ruptured plaque (original magnification, ×200.). The area of the micrograph corresponds to the box shown in **B **in an adjacent section. Panel **D **depicts a higher power view of NOD2 co-localization with CD68+ macrophages in inflammatory infiltrates in the intima. (original magnification, ×50). Arrowheads indicate double-positive cells. Pictures are representative for five patients and five controls (heart beating donors).

Immunofluorescent staining of NOD2 in coronary arteries from five healthy controls (heart beating donors) and five patients with macroscopically and microscopically proven CHD showed the presence of NOD2 protein at high staining intensities in atherosclerotic plaques, especially in areas infiltrated with inflammatory cells (Fig. 1A,B). In the ruptured plaques, NOD2-positive cells were observed in the vicinity of the vascular lumen (Fig. 1C). Double staining for NOD2 and cell-type specific markers (Fig. 1C,D) indicated that NOD2 is also expressed by CD68-positive macrophages in deeper layers of the intima (arrows in Fig. 1B and 1D). von Willebrand factor-positive endothelial cells only weakly express NOD2 (data not shown), which is functionally supported by data demonstrating the MDP-responsiveness of cultured endothelial cells [19,20].

### Functional CARD15 variants are not associated with CHD

A total of 900 German early-onset CHD patients and 632 healthy control individuals were subjected to analysis of the *CARD15 *variants R702W, G908R and 1007fs. The genotype distributions of all markers were in Hardy-Weinberg equilibrium in both cases and controls. Prior to risk factor adjustment, there was no statistically significant difference between any of the three mutations and the CHD phenotype, at the allele and genotype level (Table 1). Neither stratification by survival of myocardial infarction revealed any significant association (Table 1). Since all three *CARD 15 *variants are relatively rare, we compared the frequencies of carriers (i.e. homozygote, heterozygote and double heterozygote carriers of any of the three polymorphisms) between the CHD samples and the controls. The carrier frequencies were not significantly different between the two groups (0.170 vs. 0.190; odds ratio [OR] = 0.87 with 95% confidence interval ranging from 0.67 to 1.14). A power calculation revealed that given the sample sizes and an average carrier risk factor frequency of 0.18, we would be able to detect a variant with a relative risk of 1.46 (at a significance level of 0.05 and a power of 80%). In addition, logistic regression analysis, which facilitates the adjustment of known CHD risk factors including gender, yielded no evidence for association (Table 2). Based on these results, none of the analyzed *CARD15 *variants seem to play a significant role in the etiology of CHD.

**Table 1:** *CARD15 *variants in German CHD and control samples

***CARD15 *variant**	**CHD**	**MI**	**Controls**	***P *(CHD)**	***P *(MI)**
**R702W (rs2066844)**					
Number of individuals	890	589	632		
Variant frequency	0.046	0.045	0.055	0.246	0.242
Genotype frequencies				0.173	0.290
C/C	0.913	0.914	0.890		
C/T	0.083	0.083	0.108		
T/T	0.004	0.003	0.002		

**G908R (rs2066845)**					
Number of individuals	900	596	632		
Variant frequency	0.017	0.017	0.013	0.476	0.499
Genotype frequencies				0.626	0.553
G/G	0.968	0.968	0.973		
G/C	0.031	0.030	0.027		
C/C	0.001	0.002	0.000		

**1007fs (rs2066847)**					
Number of individuals	897	594	630		
Variant frequency	0.027	0.030	0.026	0.736	0.892
Genotype frequencies				0.489	0.583
-/-	0.945	0.939	0.942		
-/insC	0.055	0.061	0.056		
insC/insC	0.000	0.000	0.002		

Abbreviations: CHD, coronary heart disease; MI, myocard infarction; insC, cytosine insertion

**Table 2:** Logistic regression analysis with adjustment for CHD confounding factors

**Model**	**Dominant**		**Additive**		**Recessive**	
**Variant**	***P***	**OR (CI)**	***P***	**OR (CI)**	***P***	**OR (CI)**
**R702W**	0.907	0.66 (0.0–104.2)	0.940	1.03 (0.5–2.0)	0.933	1.03 (0.5–2.2)
**G908R**	NA	NA	0.255	0.50 (0.2–1.7)	0.255	0.50 (0.2–1.7)
**1007fs**	0.984	NA	0.245	0.63 (0.3–1.4)	0.233	0.62 (0.3–1.4)

Logistic regression was performed under a dominant, an additive, and a recessive genetic disease model. The *P *values (*P*) are nominal (without further correction for multiple testing) and were obtained from a Wald for significant deviations of the regression parameter from 0. The odds ratio (OR) is given, including the 95% confidence interval (CI). Adjustment was done for the following confounders: gender, BMI, smoking status, diabetes status and high blood pressure status. NA, not applicable.

## Discussion

A wide spectrum of bacterial species has been detected in atherosclerotic coronary lesions, most of them compatible with commensals on surfaces of human barrier organs including the mucosa of the respiratory or gastrointestinal tract and the skin [10]. In addition, pathogens of the respiratory tract (e.g. *Chlamydia pneumoniae*) or from the gingival (e.g. *Porphyromonas gingivalis*) are consistently found in atheromas [29-31]. The inter-individual variation in the bacterial colonization of atherosclerotic lesions, the diversity of existing microbes, and the compatibility with the constitution of the microflora on human barriers raise the possibility that the pathophysiology of CHD could involve a defect of innate immunity and, therefore, barrier dysfunction. In this case, bacteria would be able to translocate from their natural habit in biofilms (e.g. on mucosal surfaces) into systemic circulation and contribute to local inflammation through colonisation of atheromatous lesions. This hypothesis is supported by epidemiologic findings that show association of rheumatoid arthritis, periodontitis and diabetes, in which an increased permeability of the oral and intestinal mucosa has been reported, with general atherosclerosis and CHD [32-36].

Receptors for pathogen associated microbial patterns (PAMPs) play a pivotal role in the innate immune defense. Expression of TLRs in coronary artery lesions, in particular TLR1, TLR2 and TLR4, is enhanced and appears to be crucial for the regulation of inflammatory activity and bacterial recognition in atheromas [12,13]. Edfeldt *et al*. found significant association between *TLR4 *variants and risk of myocardial infarction [13].

NOD2 is a major player in the host defense against bacterial invasion. Expression of NOD2, as an intracellular receptor for bacterial cell wall proteins (specifically MDP), is increased through TNF and IFN-gamma [25]. The NOD2 protein contains two N-terminal caspase recruitment domains (CARDs) as effector elements and NOD-LRRs as sensor domains for bacterial MDP. NOD2 is expressed mainly by macrophages and dendritic cells, but also by intestinal epithelial and endothelial cells [25-27]. In this study, we demonstrated that NOD2 is mainly expressed in macrophages in coronary artery lesions, suggesting that NOD2 might modulate the local immune response and infection control in atherosclerotic lesions. These novel findings support the hypothesis that NOD2 is not only a marker of general barrier dysfunction, but is also one of the important local innate immunity players in the atheroma lesion. As bacterial flora has been localized in atheroma tissue [10], NOD2 is likely involved in specific defense function against bacterial invasion. Moreover, PRRs confront not only microbes but also modified LDL, the most established risk factor for CHD [14]. A genetic deficiency in NOD2 function could therefore contribute to disease progression due to modified reaction of endogenous pathogens and/or sustain bacterial invasion and permanent microbial colonization of the atherosclerotic lesion.

Three coding polymorphisms in the *CARD15 *gene, R702W, G908R and 1007fs, confer most of the susceptibility to CD. Homozygosity and compound heterozygosity increase the risk of developing CD by as much as 40-fold [21]. With examination of these three polymorphisms much of the LD-based positional information for the *CARD15 *locus is captured [24]. The variants influence the function of the C-terminal LRR of the NOD2 protein as a sensor for MDP [25] and are likely to play a role in other chronic inflammatory diseases of barrier organs, including atopic eczema and asthma [22,23,37]. NOD2 dysfunction could therefore contribute to both, a dampened local reactivity of macrophages in CHD and an increased permeability and leakiness of the gastrointestinal barrier, permitting an enhanced entry of bacteria into systemic circulation [38]. In this study, the three main functional *CARD15 *polymorphisms were investigated in 1532 German CHD patients and healthy controls. All patients had an early onset of CHD (before age 55), and a subset of 596 patients suffered myocardial infarction as the severest phenotype of CHD. Case-control analysis revealed no statistically significant differences in the allele and genotype frequencies of the *CARD15 *variants. Neither did logistic regression analysis, which takes into consideration known CHD risk factors, yield evidence for association. Similarly, our previously reported data showed no significant association of CHD with functional mutations in the *CARD4 *gene [39]. Our finding in the present investigation is consistent with the results of a recent study in which none of the three *CARD15 *coding polymorphisms were found to influence the high rates of atherosclerotic events after renal transplantation [40].

## Conclusion

In the current study, we could demonstrate that NOD2 is up-regulated in atheroma lesions in CHD. However, genetic variants of *CARD15 *were not found to be associated with the disease in a large German sample of early-onset CHD patients. Based on these results, a major genetic contribution of *CARD15 *to the etiology of CHD can be excluded. However, other barrier function genes still remain important candidates for CHD susceptibility.

## Methods

### Recruitment of study participants

The "case" sample of 900 CHD patients comprised a population-representative collection of unrelated German coronary heart disease patients with disease onset < 55 years. They were recruited from the county of Schleswig-Holstein, the northernmost region in Germany through the population-based PopGen biobank [39,41]. In the recruitment area, all coronary angiograms of the five cardiac catheterization laboratories were screened. Study subjects were required to have coronary catheterization demonstrating significant CHD (at least a 70% stenosis in one major epicardial coronary vessel). The majority of subjects (90.3%) had a history of severe CHD and had undergone coronary revascularization procedure (percutaneous coronary intervention or coronary artery bypass grafting). A subset of 596 individuals had suffered a myocardial infarction (Table 1). The patients were between 33 and 71 years of age at the time of recruitment (mean age: 54 years) and younger than 55 years at the time of disease onset (mean age: 48 years). The gender proportions in the entire sample were 83.7% males *vs*. 16.3% females. A total of 632 healthy control individuals was also drawn from the PopGen biobank [41]. The controls were residential in the same geographic area ensuring a distribution of environmental CHD risk factors identical to those of the cases. They were randomly identified through official population registries ("Einwohnermeldeamt"), were of German ancestry (all had German parents) and ranged in age between 48 and 77 years (mean age: 62 years). Information on the general health status and the physical risk factors of CHD, i.e. smoking, body mass index (BMI), diabetes and high blood pressure, was obtained from a questionnaire that was completed during medical consultation. A subsequent clinical check-up was additionally performed. Furthermore, information on earlier CHD and myocardial infarctions was noted. Individuals who reported earlier CHD and/or myocardial infarctions were not included in the study. All participants gave informed consent prior to enrolment, and the Ethics Committee of the University Hospital Schleswig-Holstein, Campus Kiel approved the study.

### NOD2-immunostaining and fluorescence microscopy

Biopsies were embedded in cryomatrix and snap-frozen in liquid nitrogen. Cryostat sections (8 μm thick) were thaw-mounted onto Superfrost™ slides, postfixed for 5 min in acetone, airdried and stored at -20°C before further processing. Two slides of each biopsy were stained with hematoxylin-eosin for routine histological evaluation. The other slides were permeabilized by incubation with 0.1% Triton X-100 in 0.1 M phosphate-buffered saline (PBS), washed three times in PBS and blocked with 0.75% bovine serum albumin (BSA) in PBS for 20 minutes. Sections were subsequently incubated with primary antibodies (polyclonal anti-NOD-2, Cayman Chemicals, USA and monoclonal CD68, Dako, Germany) at a 1:200 dilution in 0.75% BSA for 1 h at room temperature. After washing in PBS, tissue bound antibody was detected using biotinylated goat-anti rabbit IgG antibodies (Vector) followed by FITC-conjugated avidin, both diluted at 1:100 in PBS and/or Cy3-conjugated rabbit-anti mouse (Jackson Immuno, PA, USA) at 1:350 in PBS. Controls were included using irrelevant primary antibodies as well as omitting the primary antibodies using only secondary antibodies and/or FITC-conjugated avidin. Fluorescence was detected by an Axioimager Apotome-equipped microscope (Zeiss, Germany) with appropriate filter systems and pictures were captured by a digital camera system (Axiocam, Zeiss, Germany).

### Genotyping

DNA samples from patients and control subjects were analyzed for the three major functional *CARD15 *variants R702W (rs2066844), G908R (rs2066845) and 1007fs (rs2066847) using TaqMan^® ^SNP Genotyping Assays (Applied Biosystems, Foster City, USA). The callrate of the genotypes was at least 99% for any of the three assays.

### Statistical analysis

All three mutations were assessed for significant deviation from the Hardy-Weinberg equilibrium in controls and cases using the software HaploView, version 3.2 [42]. Single marker case-control analyses were performed with χ^2 ^statistics or Fisher's exact test using the statistical genetics software GENOMIZER [43]. Power calculation was carried out with OpenEpi [44]. To allow for adjustment of the five known CHD confounding factors (gender, BMI, blood pressure, diabetes and smoking), logistic regression analysis was performed within the R statistical environment, version 2.3.1 [45].

## Authors' contributions

NE conceived of the study, drafted the manuscript and was responsible for recruitment and phenotyping of the coronary heart disease patients and the controls as part of the PopGen biobank project. SO participated in the study design and in the writing of the manuscript. AN contributed to the study design, was responsible for part of the genotyping, wrote and and critically revised the manuscript. AS performed part of the genotyping and was involved in the data analysis. PR and MF conducted the immunostaining and fluorescence microscopy. MN performed the data analysis. RS and SS directed the study. All authors approved the final manuscript.
